# Salivary nitric oxide, Simplified Oral Hygiene Index, and salivary flow rate in smokers and non-smokers: a cross-sectional study

**DOI:** 10.12688/f1000research.20099.2

**Published:** 2020-03-16

**Authors:** Endang Winiati Bachtiar, Atikah Cyntia Putri, Boy Muchlis Bachtiar

**Affiliations:** 1 Oral Biology and Oral Science Research Center, Faculty of Dentistry, Universitas Indonesia, Jakarta, DKI Jakarta, 10430, Indonesia

**Keywords:** Salivary Nitric Oxide, Oral Hygiene Index, Salivary Flow Rate, Smoker, Griess Method, Whole Saliva

## Abstract

**Background: **Salivary nitric oxide plays an important role as an antibacterial agent in the oral cavity. Here, we analyze salivary nitric oxide, Simplified Oral Hygiene Index (OHI-S) scores and the salivary flow rate in smokers and non-smokers which has not been done previously.

**Methods: **A cross sectional study included 25 smokers and 25 non-smokers. Their OHI-S results were categorized as “good,” “medium,” or “bad.” Unstimulated saliva samples were collected, and their nitric oxide concentration was measured using the Griess method.

**Results: **The salivary flow rate in smokers was lower, at 0.30 ml/minute, compared to non-smokers who had a salivary flow rate of 0.33 ml/minute. This was statistically insignificant. There was a significant difference in the concentrations of nitric oxide between smokers and non-smokers (p < 0.05). Smokers had higher concentrations than non-smokers (185.4 µM Vs 114.60 µM). In addition, there was a moderate positive correlation (r = 0.305) between the concentration of salivary nitric oxide level and the OHI-S results.

**Conclusions: **It was concluded that salivary nitric oxide concentration was higher in smokers, and the oral hygiene condition of smokers was poor.

## Introduction

Salivary nitric oxide (NO) is a free radical that plays an important role as a regulator for various physiological and pathological body mechanisms. It can act as a nonspecific antimicrobial defense system in the oral cavity, either through inhibiting bacterial growth or enhancing macrophage-mediated cytotoxicity ^1, 2^. There are two main sources of salivary NO in the oral cavity; chemically from physiological reduction by the enzyme nitrate reductase, released by certain bacteria, that converts nitrate (NO _3_-) to nitrite (NO _2_-). The other is through a reduction process facilitated by certain bacterial products resulting from the dental plaque microflora, forming nitrogen oxide and nitric acid. Unstable and spontaneous nitric acid decomposes into NO ^3^. NO can also be produced enzymatically from L-arginine, induced by nitric oxide synthase (NOS), an enzyme in the salivary gland ^2^. NOS has three isoforms: neuronal NOS (nNOS; Type I, NOS-1); inducible NOS (iNOS; Type II, NOS-2) present in various cells and tissues, produced in immunocompetent cells, like macrophages, infected by bacteria involved in regulating inflammatory reactions; and endothelial NOS (eNOS; Type III, NOS-3) found in the endothelial vascular cells ^3, 4^. High levels of NO can cause inflammation, immune disorders, neurological diseases, atherosclerosis, and cancer ^4^.

The relationship between oral health and the body's ability to produce nitric oxide is mainly part of a system known as an enterosalivary sequence. Nitric oxide (NO) levels in the oral cavity and saliva have been associated with various oral diseases. There is a hypothesis that the harmful effects of tobacco smoke may be the result of the accumulation of oxidative endothelial cell damage. Due to the presence of NO in tobacco smoke (about 500 ppm of NO and other active nitrogen oxides), it was expected that as a result of smoking, the NO metabolites in systemic circulation will increase ^5^. Since smoking may affect the oral microenvironment, in this study the analysis was performed in relation to smoking habits.

## Methods

The study groups included 25 smokers and 25 non-smokers, aged 18–24 years, studying at the Universitas Indonesia; consecutive sampling was used from August to October 2018. A sample size of convenience was used. Invitation to participate was posted on announcement boards of the Faculty of Dentistry, Universitas Indonesia, to which participants responded via mobile telephone. This cross-sectional study obtained ethical approval from the ethical research committee of the Faculty of Dentistry, Universitas Indonesia, 2018 (89/Ethical Approval/FKGUI/VII/2018). Before samples were collected, written consent (informed consent) was obtained from participants according to the guidance provided by the Ethics Committee of Faculty of Dentistry, Universitas Indonesia.

### Inclusion criteria

All participants: 18–24 years old (late adolescence/young adulthood), in good health, absence of systemic disease or history of systemic disease, normal growth and development, not having consumed food for the two hours before sampling. We assessed young adults assuming that they had not suffered any age related disease that could interfere with levels of NO. Smokers: not having smoked in the hour prior to sampling, been smoking for more than six months and consume at least one tobacco cigarette per day (containing no cloves). Non-smokers: never smoked or not smoked for more than 5 years.

### Exclusion criteria

All participants: age <18 years or >24 years, having systemic or congenital diseases related to the oral cavity, currently undergoing medical therapy, currently taking local or systemic medication that might affect saliva composition.

All participants were interviewed in order to obtain all data, which were recorded using a data form (available as *Extended data*) ^6^ prior to data entry into a computer spreadsheet. Each interview was conducted by two authors of this study team (ACP and EWB) at the Faculty of Health Science Universitas Indonesia. Depok Indonesia. Participants were instructed not to ingest food for a minimum of two hours before their saliva samples were collected. The smokers were classified into “light,” “moderate,” and “heavy” smokers based on the number of cigarettes consumed in a day (light:1–10 cigarettes, moderate; 11–20 cigarettes and heavy smoker, smoking more than 20 cigarettes), Individuals who had not smoked in the past 5 years were classified as non-smokers. According the study at the Behavioral Endocrinology Laboratory at Penn State University. Newport, one cigarette contains on average 13.4 mg nicotine. Hence we categorized in a day light smoker have consumed less than 134 mg nicotine, moderate smoker between 134–268 mg nicotine and heavy smoker more than 268 mg nicotine.

Oral hygiene index is a method for classifying the oral hygiene status of population that was determined using the Simplified Oral Hygiene Index (OHI-S), comprising two components: plaque (debris) and calculus. The OHI-S was calculated on six tooth surfaces consisting of buccal surfaces in teeth 11, 16, 26, and 31; and lingual surfaces in teeth 36 and 46 ^7^. The OHI-S categories and their corresponding scores were as follows, good (0.0-1.2) moderate (1.3-3.0) and bad (3.1-6.0) ^7^.

In this study, the saliva samples collected were unstimulated whole saliva (UWS). Over a period of three minutes, all saliva was collected in a 14-mL Falcon tube and the resulting volume was divided to 3 to get the salivary flow rate per minute. Following this, a further sample was collected by having individual participants spit 1 ml saliva into a sterile 14 ml tube. It was then transferred and stored in 1.5 ml microcentrifuge tube containing 500 µL phosphate buffer saline (PBS) (Sigma-Aldrich Dorset UK. Cat.P4417) and a protease inhibitor 0.1M Phenyl methane sulfonyl fluoride (PMSF) (Sigma-Aldrich, Cat. 329-98-6). The saliva samples were centrifuged at 5000×g for 5 min at 4°C. This was done, to separate the supernatant and the pellet in the saliva. The supernatant, containing organic components, was then stored at -20°C and the pellets were discarded. The NO concentration of the supernatant was determined by nitrite levels, using the Griess reaction (Sigma-Aldrich, Dorset, UK, Cat. G4410). Briefly, a duplicate of 50 µL of the supernatant and 50 µL of Griess reagents were mixed and incubated in the dark for 10 minutes at room temperature, then the mixture was read at 450 nm by a spectrophotometer (AccuReader. M965/M965+) Nangang, Taipei, Taiwan.

### Statistical analysis

Data were analyzed using SPSS version 22.0. The Mann-Whitney U-test was used to analyze differences between smokers and non-smokers, ANOVA was used to analyze the significant difference between four groups (non-smokers, light smokers, moderate smokers, and heavy smokers). Spearman’s correlation was used to analyze the relationship between salivary flow rate or salivary nitric oxide concentration and OHI-S (for smokers only).

## Results

### Unstimulated whole salivary flow rate in smokers and non-smokers

**Figure 1.**
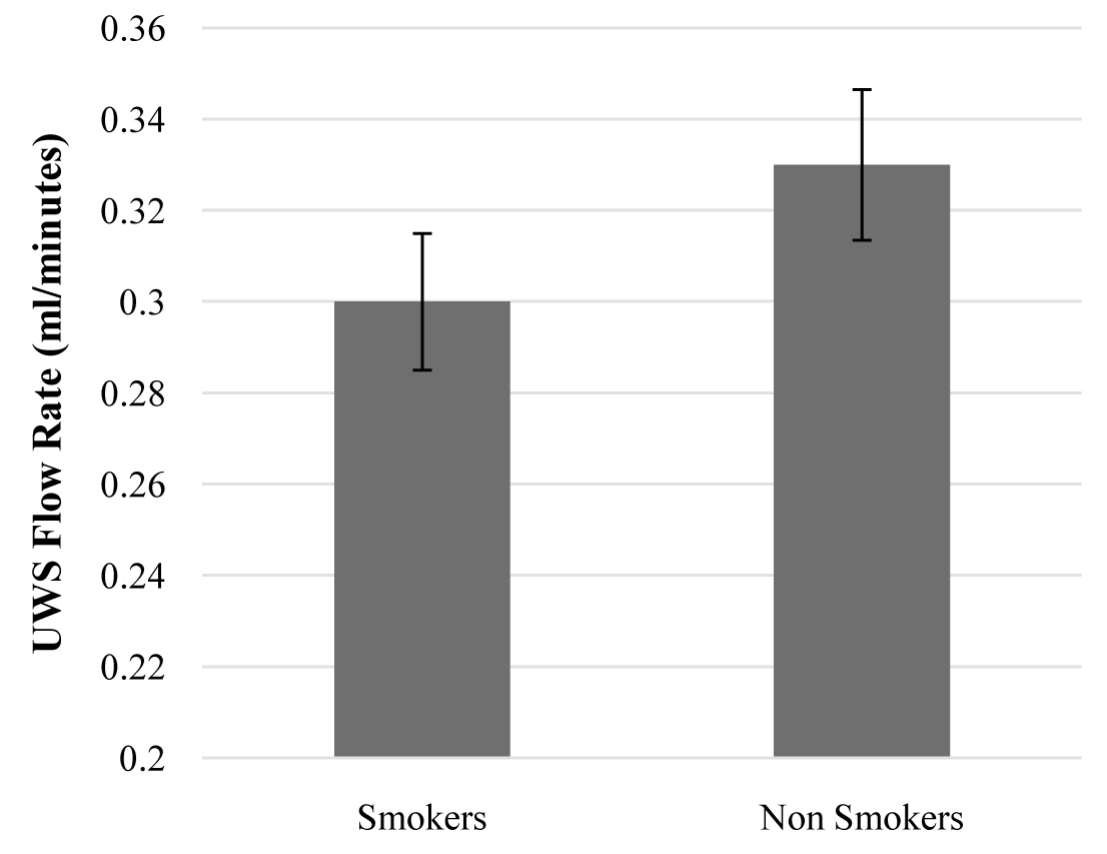
Unstimulated whole salivary (UWS) flow rate in smokers and non-smokers.

The results, after measuring the unstimulated salivary flow rate in 25 smokers and 25 non-smokers, showed that the flow rate in smokers was lower at 0.30 ml/minute, with a minimum value of 0.10 ml/minute and a maximum value of 1.67 ml/minute. In comparison, non-smokers had a flow rate of 0.33 ml/minute, with a minimum value of 0.06 ml/minute and a maximum value of 1 ml/minute ( Figure 1). Analysis through the Normality test showed that the distribution of data was abnormal. There was no significant difference between unstimulated salivary flow rates in smokers and non-smokers according to the Mann-Whitney statistical test (p = 0.748).

### Unstimulated whole salivary flow rate within the smokers’ group, based on the extent of smoking

**Figure 2.**
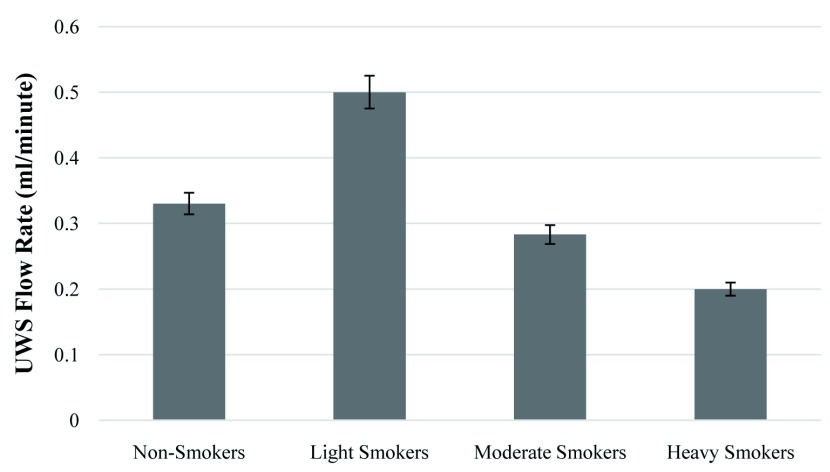
Unstimulated whole salivary (UWS) flow rate within smokers’ group based on extent of smoking.

There was a trend of decreasing salivary flow rate with increasing levels of smoking from “light,” to “moderate,” to “heavy”. The results of the Kruskal Wallis statistical test showed that the average unstimulated salivary flow rate in heavy smokers was lower than that of light and moderate smokers, with no significant difference between the smokers (p = 0.151). As shown in Figure 2, light smokers had an unstimulated salivary flow rate of 0.50 ml/minute (minimum value of 0.13 ml/minute and a maximum of 1.67 ml/minute). The unstimulated salivary flow rate in moderate smokers was 0.2830 ml/minute (minimum 0.10 ml/minute, and maximum value was 0.67 ml/minute). Heavy smokers had a salivary flow rate of 0.20 ml/minute with a minimum value of 0.10 ml/minute and a maximum value of 0.30 ml/minute.

### The concentration of salivary nitric oxide in smokers and non-smokers

**Figure 3.**
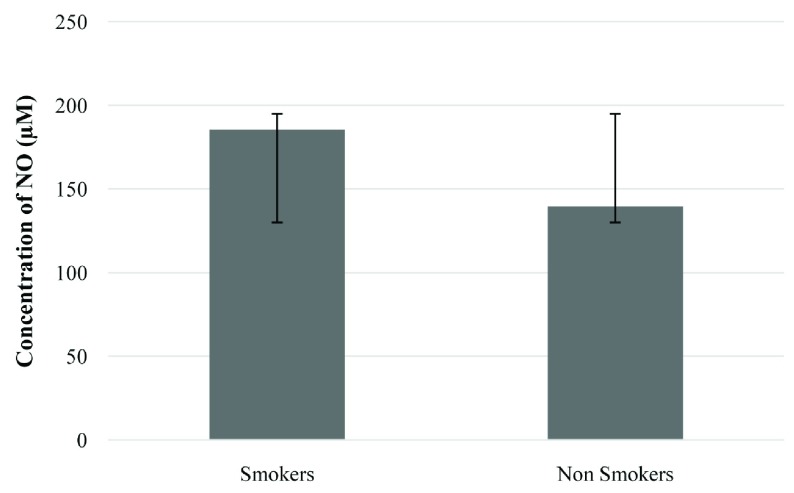
Salivary nitric oxide concentration in smokers and non-smokers.

Based on the results of the Mann-Whitney statistical test, there was a significant difference between the concentrations of NO in smokers and non-smokers (p = 0.040). Smokers displayed higher salivary NO concentrations (185.4 µM; 114.60-295.10 µM) than non-smokers (139.5 µM; 97.70-538.4 µM) ( Figure 3).

### The concentration of salivary nitric oxide within the smokers’ group, based on the extent of smoking

**Figure 4.**
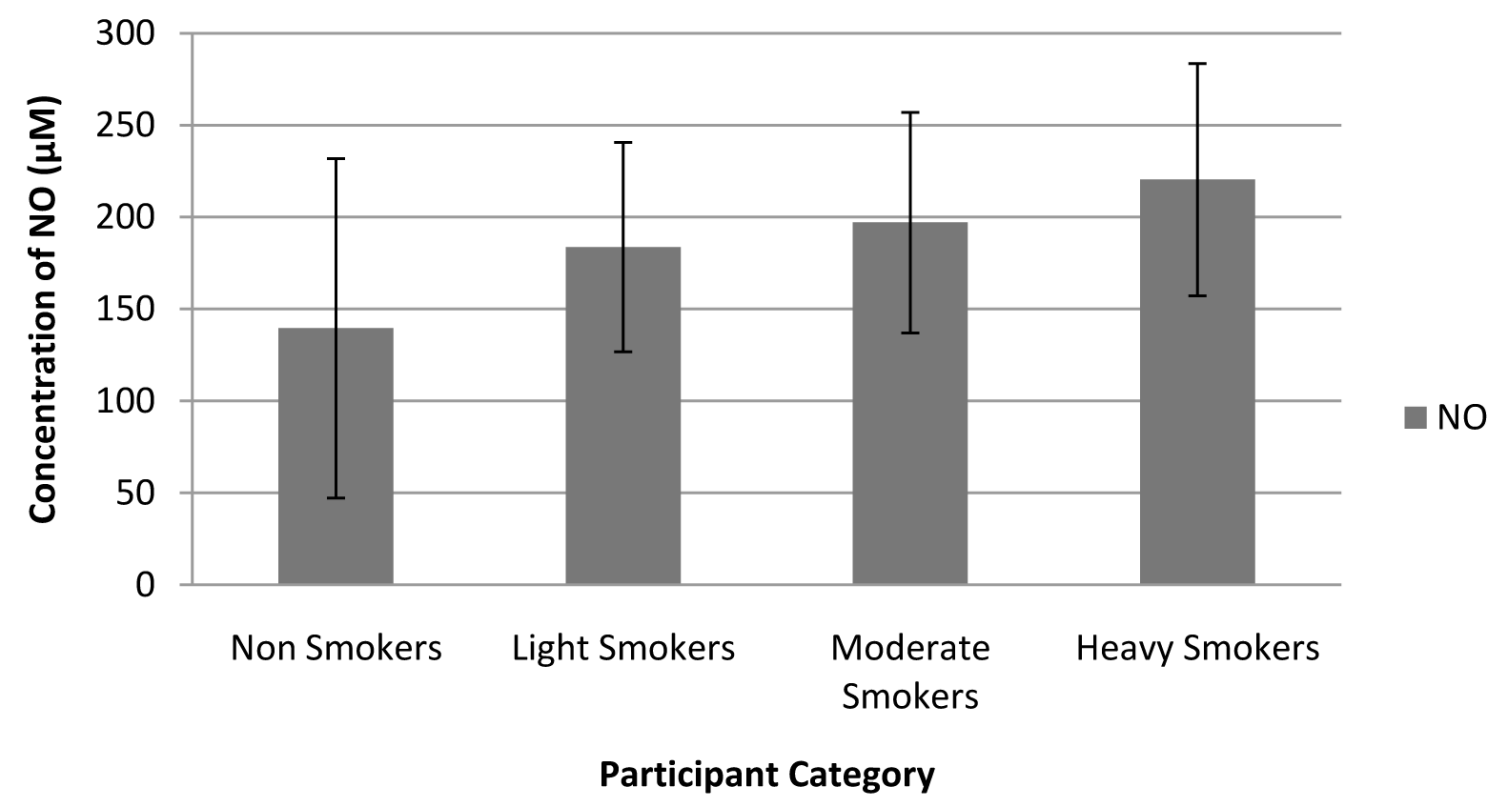
Salivary nitric oxide concentration within the smokers’ group, based on the extent of smoking.

Amongst the 25 smokers, 14 (56%) were light smokers, 8 (36%) were moderate smokers, and 3 (12%) were heavy smokers. Figure 4 shows an increasing trend in NO concentrations in the saliva with increased extent of smoking. ANOVA analysis found no significant difference in NO concentration (p = 0.600) between the different smoker groups. The concentration of NO in heavy smokers was higher than that for moderate and light smokers; 220.333 µM for heavy smokers, 196.950 µM for moderate smokers, and 183.642 µM for light smokers.

Relationship between unstimulated whole salivary flow rate and the status of oral hygiene indicated by the OHI-S for the smoker group.

**Figure 5.**
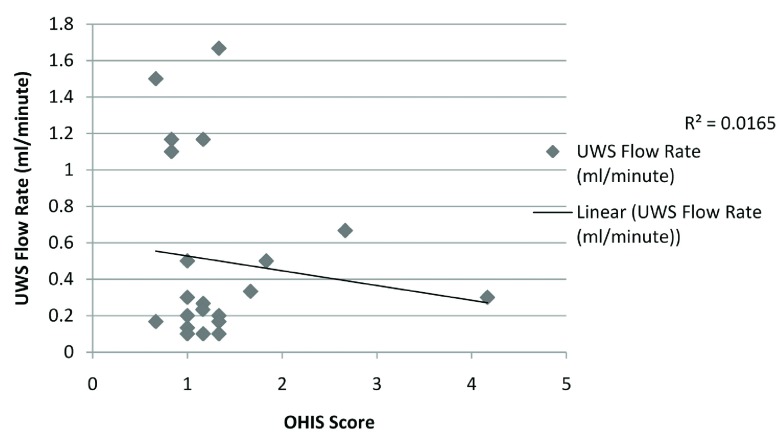
Relationship between salivary flow rate and oral hygiene status in the smokers group. UWS, unstimulated whole saliva.

Results of the Spearman’s correlation test to determine the relationship between salivary flow rate of the smokers and the status of oral and dental hygiene indicated by OHI-S scores, found an insignificant negative relationship (r = -0.118, p = 0.573). This is depicted in Figure 5.

### The relationship between salivary nitric oxide concentration and the status of oral hygiene assessed through the OHI-S of smokers

**Figure 6.**
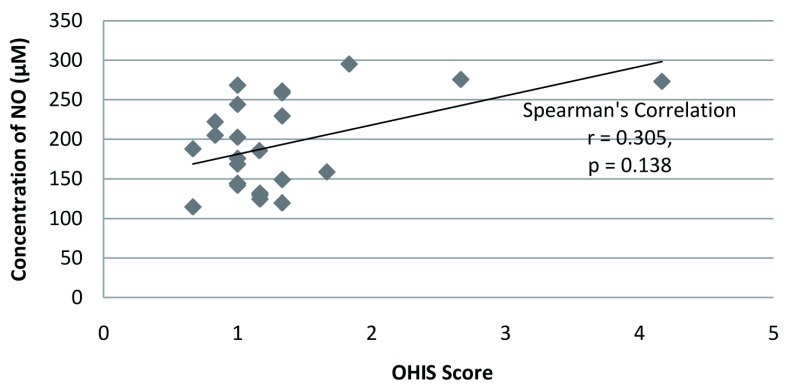
Relationship between salivary NO concentration and oral hygiene status in the smokers’ group.

The results of the analysis of the relationship between NO concentrations and oral hygiene status assessed through the OHI-S of smokers are illustrated in Figure 6. The Spearman’s correlation test showed that there was a moderate positive but insignificant relationship (r = 0.305, p = 0.138) between the concentration of NO and the OHI-S.

## Discussion

NO is a key element in saliva that regulates various physiological and pathological mechanisms of the body and can act as an antibacterial agent ^2, 4^. Saliva can act as a biomarker for the occurrence of health problems, especially in the oral cavity, which correlates to responses from the immune system ^8^. Using unstimulated saliva is an accurate method for analyzing salivary gland status through the salivary flow secreted per minute ^9^.

In this study, we observed smoking habits can affect NO concentration in saliva. This was further associated with dental and oral hygiene. As a result of exposure of the oral cavity to the components of cigarettes, smoking habits can affect elements in the oral cavity, one of them being the salivary flow rate ^10^. This study showed that the salivary flow rate for smokers was lower, compared to non-smokers, though the difference was not significant. The results of this study correlate with the results of Khan *et al.* which also stated the lack of a significant difference between salivary flow rates of smokers and non-smokers ^10^. According to Singh *et al.* and Khan *et al.*, there is a decrease in the flow rate of saliva in smokers compared to non-smokers owing to the heat and effect of toxins from cigarettes produced during smoking. There is a declined blood flow in the salivary glands, causing decreased flow of saliva from the disturbed salivary gland ^9, 10^.

The process of salivary secretion is quite complex, and the flow and composition of saliva can be influenced by several factors ^11^. These include the duration and extent of smoking per day, the more cigarettes consumed and longer the duration of the oral cavity’s exposure to the content of cigarettes, the lower the flow of saliva in the mouth ^12, 13^. Previous studies reported that heavy smokers who indulged in regular smoking habits for more than 6 months, showed decreased salivary secretions compared to non-smokers ^9, 13^. In a study conducted by Fenoll-Palomares *et al*., there was an increase in salivary flow rate in smokers who smoked a short period, but the difference between salivary flow rates in long-term smokers and non-smokers was unknown ^14^. In this study, as with the previous studies, the salivary flow rate of heavy smokers was lower than light and moderate smokers, indicating that smoking intensity affects the secretion of salivary flow rates. Data from Figure 2 indicates that light smokers can experience increased salivary gland activity leading to an increase in the rate of saliva flow, while those who have smoked for a longer period of time decrease the rate of saliva flow ^12^.

The reduced flow rate of saliva can affect its protective function in the oral cavity. The content of cigarettes, can cause poor oral hygiene, halitosis, and lead to reddish and white local lesions ^15^. Based on the results of this study, we found a weak negative relationship between salivary flow rates in smokers, and dental and oral hygiene conditions. Several studies have shown that oral hygiene conditions in patients who are smokers are worse in regard to dental plaque accumulation, compared to those who are non-smokers ^16, 17^. This is due to smoking habits that affect salivary flow rates as the parotid gland’s function of secreting dilute saliva, is adversely affected when exposed to cigarette smoke. This causes a change in the nature of the saliva secreted. For instance, the sublingual and submandibular glands will secrete mucin saliva, having a thicker consistency, increased salivary mucin can reduce other components of saliva such as immunoglobulins and enzymes, which have function as an inhibitor of plaque growth and so a reduced quantity of saliva can increase plaque formation ^18, 19^.

Changes in salivary flow rate due to smoking can affect dental and oral hygiene. The study by Dawes and Watanabe reveals that reducing the stimulated salivary flow rate, which retards the rate of salivary clearance of sugar from the oral cavity ^20^. Decreasing of the clearance of sugar might be followed by a decrease of OHIS. The increase of salivary flow is much more effective in neutralizing and buffering food acids. The study by Shimazaki *et al*. reveals that decreased saliva secretion affects both dental caries and general periodontal health status ^21^. Ahmad *et al.* also reported that hyposalivation due to the low saliva flow rate related to more caries in the aging population and significantly poor of the quality of life ^22^. The physiological balance inside the oral cavity is disturbed due to smoking, demonstrated by the concentration of NO in the saliva ^23^. This study found that NO concentrations in smokers were significantly higher than non-smokers. This, however, contrasts with the findings of Rezaei *et al.*, where the concentration of NO in smokers was lower than that of non-smokers due to a decrease in the expression of the eNOS enzyme gene following exposure to cigarettes, causing decreased NO metabolism ^1^. NO is produced from NO-forming enzymes, additional dietary or atmospheric NO intake, and the result of bacterial oxidation metabolism, which acts as a non-specific defense mechanism in the oral cavity to prevent excessive bacterial growth ^19, 24^. In this study, the increased concentration of NO was likely due to high bacterial activity, causing plaque, indicated by the high OHI-S.

The results of this study from the correlation tests conducted by the researchers reveal that there was a moderate positive but insignificant relationship between salivary NO concentration and the status of dental and oral hygiene, assessed using the OHI-S. This study is in line with the research conducted by Mobarak and Abdallah which showed no significant relationship between the concentration level of NO and the status of oral hygiene ^25^. According to Bayindir *et al*., increased NO concentration correlated with poor oral and dental hygiene ^26^. This is because the thickness of the plaque that is mature, inhibits the diffusion of oxygen into the deeper layers where the facultative anaerobic bacteria are located. Resultantly, the bacteria multiply owing to lack of oxygen, producing more NO because of increased bacterial activity.

The results of this study seem to indicate that in smokers if OHI-S increases it is followed by an increase in NO levels in saliva. This phenomenon may be due to an increase in plaque accumulation which might trigger NO production.

The potential bias in this study is the possibility of determining smoking frequency because it is only based on interviews. This research also has limitations in the number of samples because within the timeframe specified for recruiting participants only certain people are willing to participate as research subjects. The possible limitations for using Falcon tubes in saliva collection in this study might interfere with the results of NO measurement. The ideal methods in collecting saliva ^27^ should be applied for future research in analyzing another salivary biomarker to overcome the limitation of this study. The results of this study are expected to contribute to further research in order to find strategies to improve oral health in smokers.

## Data availability

### Underlying data

Open Science Framework: Salivary nitric oxide, Simplified Oral Hygiene Index, and salivary flow rate in smokers and non-smokers. https://doi.org/10.17605/OSF.IO/WD2GV ^6^.

This project contains the following underlying data:

- F1000 Raw data for NO concentrations (salivary NO concentrations of each participant).
- F1000 S-OHIS (raw data for plaque and calculus for each participant).

### Extended data

Open Science Framework: Salivary nitric oxide, Simplified Oral Hygiene Index, and salivary flow rate in smokers and non-smokers. https://doi.org/10.17605/OSF.IO/WD2GV ^6^.

This project contains the following extended data:

- Quesioner F1000 research (English translation of the questionnaire used in this study).

Data are available under the terms of the Creative Commons Zero "No rights reserved" data waiver (CC0 1.0 Public domain dedication).

## References

[1] Rezaei F Taghvai AM Vaisi-Raygani A: A Comparative Study of Salivary Nitric Oxide Between Smokers and Nonsmokers. *Sch Res Libr.* 2016;8(20):145–50. Reference Source

[2] Surdilovic D Stojanovic I Apostolovic M: The Role of Nitric Oxide in Saliva in Reduction of Caries.2008;25(2):93–5. Reference Source

[3] Wadhwa D Bey A Hasija M: Determination of levels of nitric oxide in smoker and nonsmoker patients with chronic periodontitis. *J Periodontal Implant Sci.* 2013;43(5):215–20. 10.5051/jpis.2013.43.5.215 24236243 PMC3825988

[4] Preethi S Jose JI Sivapathasundharam B: Evaluation of Salivary Nitric Oxide Levels in Smokers, Tobacco Chewers and Patients with Oral Lichenoid Reactions. *J Clin Diagn Res.* 2016;10(1):ZC63–6. 10.7860/JCDR/2016/16517.7126 26894179 PMC4740707

[5] Nene S Gelabert H Moore W: Cigarette smoking increases endothelial-derived vasorelaxation in the rat carotid artery in a dose-dependent manner. *J Surg Res.* 1997;71(2):101–6. 10.1006/jsre.1997.5147 9299276

[6] Bachtiar EW: Salivary nitric oxide, Simplified Oral Hygiene Index, and salivary flow rate in smokers and non-smokers.2019 10.17605/OSF.IO/WD2GV PMC7111499 32269757

[7] Simplified Oral Hygiene Index | OHI-S.2010 Reference Source

[8] Malamud D: Saliva as a diagnostic fluid. *Dent Clin North Am.* 2011;55(1):159–178. 10.1016/j.cden.2010.08.004 21094724 PMC3011946

[9] Chakrabarty S Patil S Bandalore SRH: A comparative study of long-term effect of tobacco on resting whole mouth salivary flow rate and pH. *J Indian Acad Oral Med Radiol.* 2015;27(4):549–52. 10.4103/0972-1363.188759

[10] Singh M Yadav P Ingle N: Effect of long-term smoking on salivary flow rate and salivary pH. *J Indian Assoc Public Heal Dent.* 2015;13(1):11–13. 10.4103/2319-5932.153549

[11] Khan GJ Javed M Ishaq M: Effect of Smoking on Salivary Flow Rate. *Gomal J Med Sci.* 2010;8(2):221–4. Reference Source

[12] Alaee A Azizi A Valaei N: The Correlation Between Cigarette Smoking and Salivary Flow Rate. *J Res Dent Maxillofac Sci.* 2017;2(3):5–9. 10.29252/jrdms.2.3.5

[13] Rad M Kakoie S Niliye Brojeni F: Effect of Long-term Smoking on Whole-mouth Salivary Flow Rate and Oral Health. *J Dent Res Dent Clin Dent Prospects.* 2010;4(4):110–4. 10.5681/joddd.2010.028 23346336 PMC3429961

[14] Fenoll-Palomares C Muñoz-Montagud JV Sanchiz V: Unstimulated salivary flow rate, pH and buffer capacity of saliva in healthy volunteers. *Rev Esp Enferm Dig.* 2004;96(11):773–83. 10.4321/s1130-01082004001100005 15584851

[15] Rehan F Khan RB Khurshid Z: Analysis of Resting Mouth Salivary Flow Rate and Salivary pH of Tobacco Chewers and Smokers. *JPDA J Pak Dent Assoc.* 2016;25(254):158–63. Reference Source

[16] Pereira ADFV Castro ACS Ramos QDL: Effects of Cigarette Smoking on Oral Hygiene Status. *J Dent Sci.* 2013;28(1):4–7. Reference Source

[17] Olusegun S Ayanbadejo P Savage KW: Oral Hygiene Status and Periodontal Treatment Needs of Nigerian Male Smokers. *TAF Prev Med Bull.* 2010;9(2):107–12. Reference Source

[18] Alfianur N Suryana B: Pengaruh Viskositas Saliva Terhadap Pembentukan Plak Gigi. *Insidental.* 2014;1(1). Reference Source

[19] Petrušić N Posavac M Sabol I: The Effect of Tobacco Smoking on Salivation. *Acta Stomatol Croat.* 2015;49(4):309–15. 10.15644/asc49/4/6 27688415 PMC4945334

[20] Dawes C Watanabe S: The effect of taste adaptation on salivary flow rate and salivary sugar clearance. *J Dent Res.* 1987;66(3):740–4. 10.1177/00220345870660030701 3475307

[21] Shimazaki Y Fu B Yonemoto K: Stimulated salivary flow rate and oral health status. *J Oral Sci.* 2017;59(1):55–62. 10.2334/josnusd.16-0372 28049967

[22] Ahmad MS Bhayat A Zafar MS: The Impact of Hyposalivation on Quality of Life (QoL) and Oral Health in the Aging Population of Al Madinah Al Munawarrah. *Int J Environ Res Public Health.* 2017;14(4): pii: E445. 10.3390/ijerph14040445 28425972 PMC5409645

[23] Bodis S Haregewoin A: Evidence for the release and possible neural regulation of nitric oxide in human saliva. *Biochem Biophys Res Commun.* 1993;194(1):347–50. 10.1006/bbrc.1993.1826 8333849

[24] Hegde MN Kumari S Hegde N: Evaluation of The Status of Salivary Nitric Oxide in Patients with Dental Caries. *J Heal Sci.* 2012;2(2):6–9. 10.1055/s-0040-1703562

[25] Mobarak EH Abdallah DM: Saliva Nitric Oxide Levels in Relation to Caries Experience and Oral Hygiene. *J Adv Res.* 2011;2(4):357–62. 10.1016/j.jare.2011.05.005

[26] Bayindir YZ Polat MF Seven N: Nitric oxide concentrations in saliva and dental plaque in relation to caries experience and oral hygiene. *Caries Res.* 2005;39(2):130–3. 10.1159/000083158 15741725

[27] Khurshid Z Zohaib S Najeeb S: Human Saliva Collection Devices for Proteomics: An Update. *Int J Mol Sci.* 2016;17(6): pii: E846. 10.3390/ijms17060846 27275816 PMC4926380

