# Supplementary material for: Salivary nitric oxide, Simplified Oral Hygiene Index, and salivary flow rate in smokers and non-smokers: a cross-sectional study
Source: F1000Res. 2020 Mar 16;8:1744. Originally published 2019 Oct 11. [Version 2] doi: 10.12688/f1000research.20099.2 (PMC7111499; doi:10.12688/f1000research.20099.2)
Supplement: Peer review history

# Peer review: Reviewer response for version 2

**DOI:** 10.5256/f1000research.25183.r61367

**Published:** 2020-03-30

**Authors:** Muhammad Sohail Zafar

**Affiliations:** 1 Department of Restorative Dentistry, College of Dentistry, Taibah University, Medina, Saudi Arabia

Many thanks to authors for revising the manuscript and responding to the previous comments.

I confirm that I have read this submission and believe that I have an appropriate level of expertise to confirm that it is of an acceptable scientific standard.

# Peer review: Reviewer response for version 2

**DOI:** 10.5256/f1000research.25183.r61368

**Published:** 2020-03-23

**Authors:** Juni Handajani

**Affiliations:** 1 Department of Oral Biology, Faculty of Dentistry, Universitas Gadjah Mada, Yogyakarta, Indonesia

Author have already added some references about correlation between salivary flow rate and oral hygiene (Dawes and Watanabe, 1987; Shimazaki et al., 2017; Ahmad et al, 2017)

I confirm that I have read this submission and believe that I have an appropriate level of expertise to confirm that it is of an acceptable scientific standard.

# Peer review: Reviewer response for version 1

**DOI:** 10.5256/f1000research.22067.r60322

**Published:** 2020-03-04

**Authors:** Muhammad Sohail Zafar

**Affiliations:** 1 Department of Restorative Dentistry, College of Dentistry, Taibah University, Medina, Saudi Arabia

I have reviewed the manuscript “Salivary nitric oxide, Simplified Oral Hygiene Index, and salivary flow rate in smokers and non-smokers: a cross-sectional study” submitted to “F1000Research” for publication. I found this work interesting and fit well with in the scope of this journal. In this cross-sectional study, authors have analyzed and compared salivary nitric oxide, Simplified Oral Hygiene Index (OHI-S) scores and the salivary flow rate in smokers and non-smokers.

 The manuscript fits well within the scope of the journal; it needs some major improvements; there are a few suggestions that authors may consider to improve it further:

- Abstract: is structured and has been described clearly; however, there is no description of statistical analysis that should be added to the abstract.
- Introduction; is brief however covering the background information and the rationale of the study effectively.
- Methodology and results have been reasonably presented; OHIS score should be defined.
- Figure 1 and 2: SFR legend is not required and must be deleted to match the style of figure 3.
- Figure 5 and 6: Use same style to describe OHIS; either OHIS or OHI-S?
- The discussion is a little bit brief; authors must include and cite more studies in context comparing their findings with previous studies. For example; why specific saliva collection devices were not used and what are the possible limitations for using Falcon tubes? The authors could include the following paper with relevant information:
  Khurshid, Zohaib, et al. "Human saliva collection devices for proteomics: An update." *International journal of molecular sciences* 17.6 (2016): 846. ^1^
- Similarly, how the reduced salivary flow and age may affect the OH and quality of life should be discussed; the information from the following papers could be included:
  Ahmad, Mohammad S., et al. "The impact of hyposalivation on quality of life (QoL) and oral health in the aging population of Al Madinah Al Munawarrah." *International journal of environmental research and public health* 14.4 (2017): 445. ^2^
  Khurshid, Zohaib, et al. "Human saliva and its role in oral & systemic health." *JPDA* 25.04 (2016): 171.
- What are the final conclusions of this study? The key outcome points should be highlighted

I confirm that I have read this submission and believe that I have an appropriate level of expertise to confirm that it is of an acceptable scientific standard, however I have significant reservations, as outlined above.

## References

[1] : Human Saliva Collection Devices for Proteomics: An Update. *Int J Mol Sci*.2016;17(6) : 10.3390/ijms17060846 10.3390/ijms17060846 27275816PMC4926380

[2] : The Impact of Hyposalivation on Quality of Life (QoL) and Oral Health in the Aging Population of Al Madinah Al Munawarrah. *International Journal of Environmental Research and Public Health*.2017;14(4) : 10.3390/ijerph14040445 10.3390/ijerph14040445 PMC540964528425972

# Response

**Published:** 2020-03-10

**Authors:** Boy Muchlis Bachtiar

**Affiliations:** Universitas Indonesia, Indonesia

Dear Reviewer,

I am pleased to resubmit for publication the revised version of Salivary nitric oxide, Simplified Oral Hygiene Index, and salivary flow rate in smokers and non-smokers: a cross-sectional study

We appreciate all the insightful comments provided by the reviewer. Based on the reviewer’s guidance, includes a number of positive changes, we endeavored to improve the fit of the paper with the journal.

We hope that these revisions improve the paper such that Editor and the Reviewer now deem it worthy of publication in F1000 Research. Thank you for taking the time to help us improve the paper.

Sincerely,

Authors :  1. Endang W Bachtiar

                2.  Atikah Cyntia Putri

                3. Boy M Bachtiar

Comment provided by Reviewer #2

I have reviewed the manuscript “Salivary nitric oxide, Simplified Oral Hygiene Index, and salivary flow rate in smokers and non-smokers: a cross-sectional study” submitted to “F1000Research” for publication. I found this work interesting and fit well with in the scope of this journal. In this cross-sectional study, authors have analyzed and compared salivary nitric oxide, Simplified Oral Hygiene Index (OHI-S) scores and the salivary flow rate in smokers and non-smokers.

The manuscript fits well within the scope of the journal; it needs some major improvements; there are a few suggestions that authors may consider to improve it further:

Abstract: is structured and has been described clearly; however, there is no description of statistical analysis that should be added to the abstract.

Introduction; is brief however covering the background information and the rationale of the study effectively.

Methodology and results have been reasonably presented; OHIS score should be defined.

Figure 1 and 2: SFR legend is not required and must be deleted to match the style of figure 3.

Figure 5 and 6: Use same style to describe OHIS; either OHIS or OHI-S?

The discussion is a little bit brief; authors must include and cite more studies in context comparing their findings with previous studies. For example; why specific saliva collection devices were not used and what are the possible limitations for using Falcon tubes? The authors could include the following paper with relevant information:

Khurshid, Zohaib, et al. "Human saliva collection devices for proteomics: An update." International journal of molecular sciences 17.6 (2016): 846.1

Similarly, how the reduced salivary flow and age may affect the OH and quality of life should be discussed; the information from the following papers could be included:

Ahmad, Mohammad S., et al. "The impact of hyposalivation on quality of life (QoL) and oral health in the aging population of Al Madinah Al Munawarrah." International journal of environmental research and public health 14.4 (2017): 445.2

Khurshid, Zohaib, et al. "Human saliva and its role in oral & systemic health." JPDA 25.04 (2016): 171.

What are the final conclusions of this study? The key outcome points should be highlighted

**Author’s response**:

1. Line 60: OHIS score has been defined.
2. Figure 1 and 2: SFR legend has been deleted.
3. Figure 5 and 6: we use OHIS.
4. Discussion section has been revised according to the reviewer suggestion and the suggested references have been added. (Line 193-200, line 227-230).
5. We have stated the final conclusion in Line 220-222

# Peer review: Reviewer response for version 1

**DOI:** 10.5256/f1000research.22067.r60176

**Published:** 2020-03-03

**Authors:** Juni Handajani

**Affiliations:** 1 Department of Oral Biology, Faculty of Dentistry, Universitas Gadjah Mada, Yogyakarta, Indonesia

This study analyzed Salivary nitric oxide, Simplified Oral Hygiene Index, and salivary flow rate in smokers and non-smokers: a cross-sectional study. The results of the study explained the relationship between unstimulated whole salivary flow rate and the status of oral hygiene also the relationship between salivary nitric oxide concentration and the status of oral hygiene assessed through the OHI-S of smokers but the conclusions do not explain the correlation as mentioned.

 This paper is good and is an important addition to the literature. The concept of correlation between salivary nitric oxide and the status of oral hygiene index is written clearly. I would like to give the suggestion of adding explanations about why the authors correlated flow rate saliva and status of oral hygiene or add literature that explains about the effect oral hygiene status on salivary flow rate.

I confirm that I have read this submission and believe that I have an appropriate level of expertise to confirm that it is of an acceptable scientific standard, however I have significant reservations, as outlined above.

# Response

**Published:** 2020-03-10

**Authors:** Boy Muchlis Bachtiar

**Affiliations:** Universitas Indonesia, Indonesia

Dear Reviewer

 I am pleased to resubmit for publication the revised version of Salivary nitric oxide, Simplified Oral Hygiene Index, and salivary flow rate in smokers and non-smokers: a cross-sectional study

 We appreciate all the insightful comments provided by the reviewer. Based on the reviewer’s guidance, includes a number of positive changes, we endeavored to improve the fit of the paper with the journal.

 We hope that these revisions improve the paper such that Editor and the Reviewer now deem it worthy of publication in F1000 Research. Thank you for taking the time to help us improve the paper.

 Sincerely,

 Authors :  1. Endang W Bachtiar

                 2.  Atikah Cyntia Putri

                 3. Boy M Bachtiar

 Author’s response to reviewer comments

 Comment provided by Reviewer #1

 This study analyzed Salivary nitric oxide, Simplified Oral Hygiene Index, and salivary flow rate in smokers and non-smokers: a cross-sectional study. The results of the study explained the relationship between unstimulated whole salivary flow rate and the status of oral hygiene also the relationship between salivary nitric oxide concentration and the status of oral hygiene assessed through the OHI-S of smokers but the conclusions do not explain the correlation as mentioned.

 This paper is good and is an important addition to the literature. The concept of correlation between salivary nitric oxide and the status of oral hygiene index is written clearly. I would like to give the suggestion of adding explanations about why the authors correlated flow rate saliva and status of oral hygiene or add literature that explains about the effect oral hygiene status on salivary flow rate.

 Author’s response:

 We thank the reviewer for the suggestions.

 As suggested by the reviewer, we have added sentence (line 193-200): Changes in salivary flow rate due to smoking can affect dental and oral hygiene.The study by Dawes and Watanabe reveals that reducing the stimulated salivary flow rate, which retards the rate of salivary clearance of sugar from the oral cavity. ^20^Decreasing of the clearance of sugar might be followed by a decrease of OHIS.The increase of salivary flow is much more effective in neutralizing and buffering food acids. The study by Shimazaki et al. reveals that decreased saliva secretion affects both dental caries and general periodontal health status. ^21^ Ahmad et al also reported that hyposalivation due to thelow saliva flow rate related to more caries in the aging population and significantly poor of the quality of life. ^22^
